# Prevalence of Structural Heart Diseases Detected by Handheld Echocardiographic Device in School-Age Children in Iran: The SHED LIGHT Study

**DOI:** 10.5334/gh.1121

**Published:** 2022-06-15

**Authors:** Saeid Hosseini, Niloufar Samiei, Avisa Tabib, Hooman Bakhshandeh, Yousef Rezaei, Mozhgan Parsaee, Fariba Rashidi Ghader, Maryam Moradian, Maryam Shojaeifard, Zahra Khajali, Sepideh Taghavi, Nasim Naderi, Golnar Mortaz Hejri, Homa Ghaderian, Golnaz Houshmand, Raheleh Kaviani, Melody Farrashi, Marzieh Pakbaz, Hoda Mombeini, Sedigheh Saedi, Alireza Alizadeh Ghavidel, Gholamreza Omrani, Ahmad Mohebbi, Mohammad Mehdi Peighambari, Mohammad Rafie Khorgami, Akbar Nikpajouh, Ahmad Amin, Majid Maleki, Carlos A. Mestres, Luigi Paolo Badano, Feridoun Noohi, On Behalf Of The Shed Light Investigators

**Affiliations:** 1 Heart Valve Disease Research Center, Rajaie Cardiovascular Medical and Research Center, Iran University of Medical Sciences, Tehran, IR; 2 Echocardiography Research Center, Rajaie Cardiovascular Medical and Research Center, Iran University of Medical Sciences, Tehran, IR; 3 Rajaie Cardiovascular Medical and Research Center, Iran University of Medical Sciences, Tehran, IR; 4 Cardiovascular Intervention Research Center, Rajaie Cardiovascular Medical and Research Center, Iran University of Medical Sciences, Tehran, IR; 5 Department of Cardiac Surgery, University Hospital Zurich, Zurich, CH; 6 Istituto Auxologico Italiano, IRCCS, Milan, Italy; 7 Department of Medicine and Surgery, University of Milano-Bicocca, Milan, IR

**Keywords:** Echocardiography, Screening, Structural heart disease, Prevalence

## Abstract

**Background:**

Structural heart disease (SHD) has great impacts on healthcare systems, creating further public health concerns. Proper data are scant regarding the magnitude of the affected population by SHD.

**Objectives:**

This study aimed to determine the prevalence of SHD among children and adolescents in an Iranian population.

**Methods:**

In this population-based study, a multistage cluster-random sampling was used to choose schools from the Tehran urban area. All students were examined using a handheld Vscan device by echocardiographer, and the results were concurrently supervised and interpreted by cardiologists. All the major findings were reevaluated in hospital clinics.

**Results:**

Of 15,130 students (6–18 years, 52.2% boys) who were examined, the prevalence of individuals with congenital heart disease (CHD) and cardiomyopathy was 152 (10.046 per 1,000 persons) and 9 (0.595 per 1,000 persons), respectively. The prevalence of definite and borderline rheumatic heart disease (RHD) was 30 (2 per 1,000 persons) and 113 (7.5 per 1,000 persons), correspondingly. Non-rheumatic valvular heart disease (VHD) was also detected in 465 (30.7 per 1,000 persons) students. Of all the pathologies, only 39 (25.6%) cases with CHD and 1 (0.007%) cases with RHD had already been diagnosed. Parental consanguinity was the strongest predictor of CHD and SHD (odds ratio [OR]: 1.907, 95% CI, 1.358 to 2.680; P < 0.001 and OR, 1.855, 95% CI, 1.334 to 2.579; P < 0.001, respectively). The female sex (OR, 1.262, 95% CI, 1.013 to 1.573; P = 0.038) and fathers’ low literacy (OR, 1.872, 95% CI, 1.068 to 3.281; P = 0.029) were the strongest predictors of non-rheumatic VHD and RHD, correspondingly.

**Conclusions:**

The implementation of echocardiographic examinations for detecting SHD among young population is feasible which detected SHD prevalence in our population comparable to previous reports. Further studies are required to delineate its economic aspects for community-based screening.

## Introduction

The early detection of Structural Heart Disease (SHD) is of great importance in children for the implementation of therapeutic modalities and close follow-ups. Previous prospective studies have revealed that the early detection of such a phenomenon can help formulate a better plan of care and improve the quality of life and outcomes of patients [1,2].

The diagnosis of congenital heart disease (CHD) has been evolved during the past decade, leading to an increased prevalence of the disease [3]. In addition, due to a paucity of data about the survival rates of patients with CHD and the improper detection of CHD, the true prevalence of CHD in the living population is unclear [4]. The prevalence of the different types of valvular heart disease (VHD) and cardiomyopathy is also unclear in children, except for a few reports in limited populations [5,6,7]. Apropos of VHD, the prevalence of rheumatic heart disease (RHD) detected by echocardiographic examinations varies among populations at low or high risk of the disease [8]. However, timely and early screening via echocardiography has resulted in a dramatic rise in the prevalence of RHD among the healthy population, underscoring the paramount role of echocardiographic screening for the early detection of RHD [9].

The high weight of regular echocardiography machines precludes their use in out-of-hospital settings; nonetheless, the emergence of the pocket-sized ultrasound devices has provided a promising screening tool in community-based investigations [10,11]. Herein, in this large-scale study, we sought to determine the prevalence of SHD in a cohort of a school-age population in Tehran, Iran, via echocardiography. Moreover, for detection of other cardiovascular diseases and risk factors, we evaluated their blood pressure, electrocardiography, and anthropometric values.

## Methods

### Study protocol and population selection

In the present prospective study, a population-based survey was conducted in Tehran, Iran, between October 2017 and December 2018. Via the multistage cluster sampling method, seven districts of Tehran were selected, and then 46 schools were chosen for the study (appendix material online). In addition to the consent form, some familial features were collected from the parents in prepared questionnaires. The study complies with the Declaration of Helsinki, and the study protocol was evaluated and approved by the Ethics Committee of the National Committee for Ethics in Biomedical Research. Further details are provided in the appendix material online.

### Anthropomorphic measurements

All examinations were carried out on-site in a standard schoolroom, which was quiet, had an appropriate ambient temperature, and featured a bed for physical examinations aside from the standard classroom objects. Height, weight, waist circumference, blood pressure, and pulse oximetry oxygen saturation were assessed before echocardiographic examinations. For the measurement of arterial oxygen saturation, pulse oximetry was performed using a handheld Care Vision HP-110 device (Medical Supply Co, Gangwon-do, South Korea). Blood pressure was measured using an automated device (Connex ProBP 3400, Welch Allyn, USA), with the subjects in resting state, at least five minutes after entrance into the examination room.

### Echocardiographic examinations

Echocardiographic examinations were performed by certified echocardiographers who have passed an educational course of echocardiographic examination in a tertiary cardiovascular center and concurrently supervised and interpreted on-site by cardiologists. All the examinations were carried out using Vscan; a pocket-sized ultrasound device (GE Healthcare, Milwaukee, WI, USA). No limitations were considered for echocardiographic views and adjusting modalities. The echocardiographic evaluations were performed using the standards mentioned in the American Society of Echocardiography (ASE) guidelines, modified for portable machines as qualitative measurements given the lack of Doppler modality [12]. All major pathologic findings in the echocardiographic assessments by Vscan were re-evaluated in hospital clinics (S60, GE Healthcare, Horten, Norway or iE33, Philips Ultrasound, Bothell, WA, USA). For further details of echocardiographic examinations please refer to the appendix material online (method section and Appendix Table 1).

### Electrocardiographic examinations

A standard 12-lead electrocardiography (ECG) was recorded in the supine position with a unique recorder machine (CP 150 model, Welch Allyn, USA). The ECG was recorded by technician, and all ECGs were checked by cardiologists. The interpretation of traces was performed according to current international standards. Further details of electrocardiographic examinations are summarized in the appendix material online (method section and Appendix Table 1).

### Statistical analysis

The continuous variables, comprising anthropometric values and physical examination findings, were presented as the mean (± standard deviation), and all the values were compared between groups using the independent *t*-test. The categorical variables were presented as frequencies/percentages calculated per 1,000 persons and were compared between groups using the χ^2^ test. The 95% confidence interval (CI) was also reported for variables. According to the sampling method, we corrected the standard deviations and confidence intervals for our data description and statistical inferences. The positive predictive value (PPV) was calculated as ‘true positive’ divided by ‘true positive’ plus ‘false positive’ findings on echocardiographic evaluations. An echocardiographic finding was considered ‘true positive’ when it was similar in both the hand-held and standard echocardiographic evaluations. We also consider those as ‘false positive’ when hand-held finding contradicted the standard echocardiography. The multivariable logistic regression analyses were also conducted to find the predictors of the echocardiographic findings. Variables found to have a P value < 0.2 in the univariable analyses were entered into multivariable models. Of the anthropometric factors, only waist circumference was entered into the regression models. All the data were analyzed using the STATA software (StataCorp, TX, USA).

## Results

### Baseline characteristics

**Figure 1:**
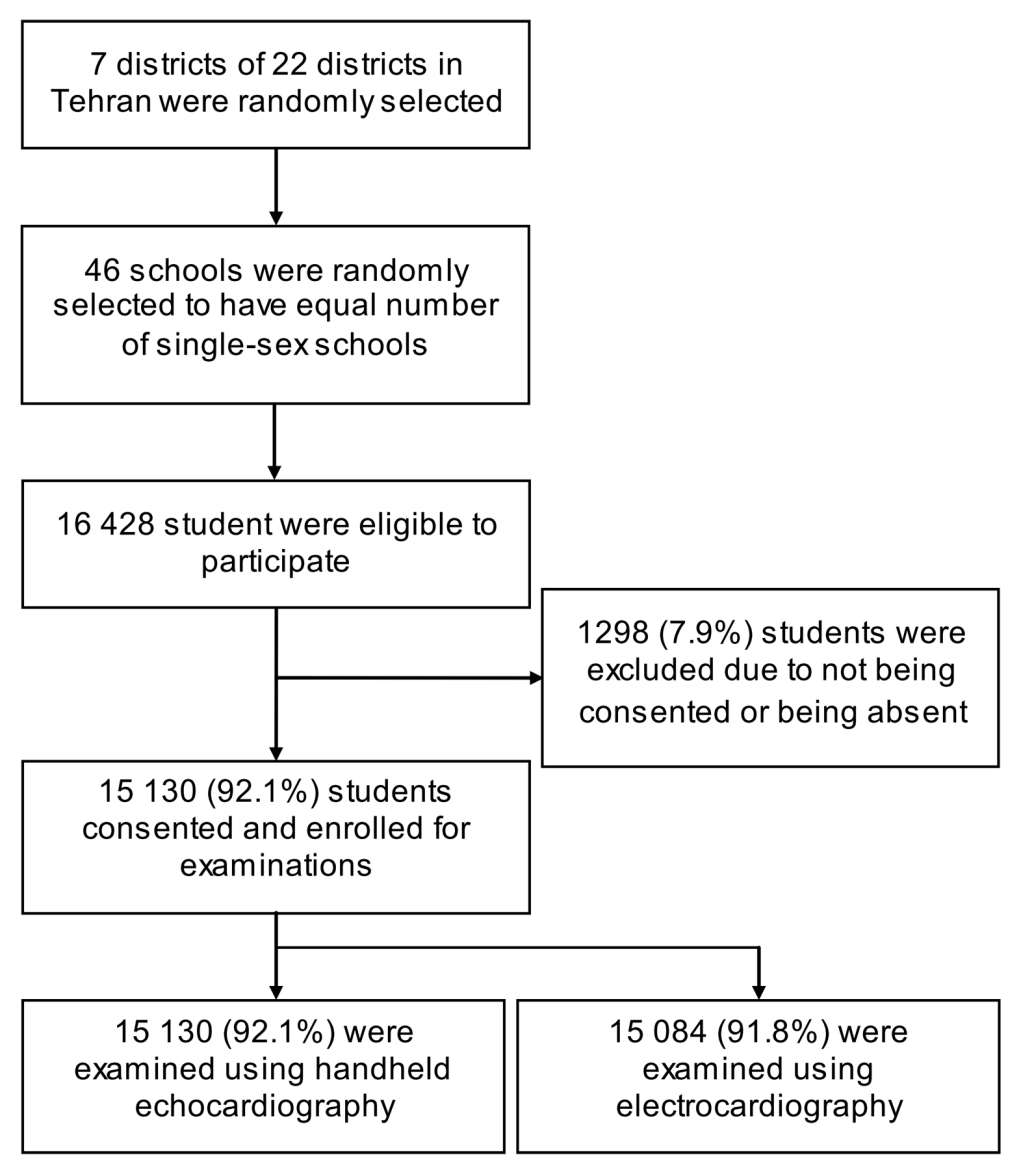
Study flow chart.

The rate of the students that signed consent forms and were fully examined was 92.1% (Figure 1). The baseline characteristics (ie, age, sex, height, weight, and parental age) of enrolled students were comparable to those of excluded ones (all p > 0.05). Therefore, a total of 15,130 students were assigned to the final analysis (13,166 [87%] in state-run schools vs 1,964 [13%] in private schools). The mean age of the students was 12.2 ± 3.1 years, and 7,894 (52.2%) were male. The rate of parental non-consent for examination was higher among girls than boys (10.6% vs 5.3%; *P* < 0.001). The distribution of the students in the age categories is illustrated in appendix figure. Other baseline characteristics are summarized in Table 1.

**Table 1:** Baseline characteristics of population.


	VALUES	95% CONFIDENCE INTERVAL

Total population	15130	…

Age, years	12.2 ± 3.1	12.1 – 12.3

Gender		

Boys	7894 (52.2%)	51.4 – 53.0

Girls	7236 (47.8%)	47.0 – 48.6

School type		

State-run	13167 (87%)	86.5 – 87.5

Private	1963 (13%)	12.5 – 13.5

Parental consanguinity	3954 (26.1%)	25.4 – 26.8

Father literacy*		

High school diploma or less	10362 (73.6%)	72.9 – 74.3

University degrees	3719 (26.4%)	25.7 – 27.1

Mother literacy*		

High school diploma or less	10867 (76.7%)	76.0 – 77.4

University degrees	3297 (23.3%)	22.6 – 24.0

Number of children in family		

1 child	3397 (22.5%)	21.8 – 23.2

2 children	7629 (50.4%)	49.6 – 51.2

3 children	2941 (19.4%)	18.8 – 20.0

≥4 children	1163 (7.7%)	7.3 – 8.1

Height,^†^ cm	151.4 ± 17.2	151.1 – 151.6

Weight,^†^ kg	48.4 ± 18.8	48.1 – 48.7

Waist circumference,^†^ cm	70.2 ± 11.4	70.1 – 70.4

Systolic blood pressure,^†^ mm Hg	113.9 ± 12.2	113.7 – 114.1

Diastolic blood pressure,^†^ mm Hg	72.3 ± 7.6	72.2 – 72.4

Arterial oxygen saturation, %	97.3 ± 1.6	97.2 – 97.4


* These data were not available in about 7% of individuals. ^†^ These data are not available in about 3% of participants. Data are presented as number (%) or mean ± SD.

### Echocardiographic screening

The median duration of echocardiographic examination by hand-held machine was six minutes (4–8). The overall prevalence of individuals with CHD detected by Vscan was 172 (11.368 per 1,000 persons; 95% CI, 9.740 to 13.188). After re-examination in hospital echocardiography clinic, the true prevalence of CHD was found to be 152 (10.046 per 1,000 persons; 95% CI, 8.519 - 11.766). Of the 152 individuals, 113 (7.468 per 1,000 persons; 95% CI: 6.2 to 9) students had not been diagnosed. All CHDs are summarized in Table 2. Ten (0.660 per 1,000 persons; 95% CI, 0.317 to 1.215) students had a combination of CHDs (Appendix Table 3).

**Table 2:** The frequency of CHDs and cardiomyopathies among students.


	NUMBERS DETECTED BY VSCAN (%)	NUMBERS DETECTED BY STE, % (95% CONFIDENCE INTERVAL)	CRUDE RATE, PER 1000 PERSONS (95% CONFIDENCE INTERVAL)

Total CHD^*^	172 (1.137%)	152, 1.005% (0.852 - 1.180)	10.046 (8.519 – 11.766)

Atrial septal defect	54 (0.357%)	50, 0.330% (0.245 – 0.435)	3.305 (2.454 – 4.355)

Patent foramen ovale	37 (0.245%)	33, 0.220% (0.150 – 0.306)	2.181 (1.502 – 3.062)

Bicuspid aortic valve	24 (0.159%)	20, 0.132% (0.080 – 0.204)	1.322 (0.808 – 2.041)

Patent ductus arteriosus	18 (0.119%)	16, 0.106% (0.060 – 0.172)	1.058 (0.605 – 1.717)

Ventricular septal defect	17 (0.112%)	16, 0.106% (0.060 – 0.172)	1.058 (0.605 – 1.717)

Coarctation of aorta	6 (0.040%)	6, 0.040% (0.015 – 0.090)	0.397 (0.145 – 0.863)

Isolated PA dilatation	5 (0.033%)	2, 0.013% (0.001 – 0.048)	0.132 (0.016 – 0.478)

Sub-aortic web	4 (0.026%)	4, 0.026% (0.007 – 0.068)	0.264 (0.072 – 0.677)

Isolated sinus valsalva dilatation	4 (0.026%)	2, 0.013% (0.001 – 0.048)	0.132 (0.016 – 0.478)

Persistent LSVC	3 (0.019%)	3, 0.020% (0.004 – 0.058)	0.198 (0.040 – 0.580)

Pulmonary valve stenosis	2 (0.013%)	2, 0.013% (0.001 – 0.048)	0.132 (0.016 – 0.478)

Isolated right aortic arch	1 (0.007%)	1, 0.007% (0.001 – 0.037)	0.070 (0.001 – 0.370)

Supravalvular mitral mass	1 (0.007%)	1, 0.007% (0.001 – 0.037)	0.070 (0.001 – 0.370)

Quadricuspid aortic valve	1 (0.007%)	1, 0.007% (0.001 – 0.037)	0.070 (0.001 – 0.370)

Aortic valve stenosis	1 (0.007%)	1, 0.007% (0.001 – 0.037)	0.070 (0.001 – 0.370)

Isolated aorta dilatation	1 (0.007%)	1, 0.007% (0.001 – 0.037)	0.070 (0.001 – 0.370)

Situs inversus	1 (0.007%)	1, 0.007% (0.001 – 0.037)	0.070 (0.001 – 0.370)

Tetralogy of Fallot	1 (0.007%)	1, 0.007% (0.001 – 0.037)	0.070 (0.001 – 0.370)

Transposition of great arteries	1 (0.007%)	1, 0.007% (0.001 – 0.037)	0.070 (0.001 – 0.370)

Other abnormalities^*^			

Redundant interatrial septum	862 (5.700%)	NA	NA

Aneurysmal interatrial septum	47 (0.310%)	NA	NA

Cardiomyopathies	32 (0.212%)^†^	9, 0.065% (0.023 – 0.113)	0.595 (0.272 – 1.129)

Idiopathic PAH^‡^	1 (0.007%)	1, 0.007% (0.001 – 0.037)	0.070 (0.001 – 0.370)


CHD denotes congenital heart disease, LSVC left superior vena cava, NA not applicable, PA pulmonary artery, PAH pulmonary arterial hypertension, and STE standard transthoracic echocardiography. * All pathologies included those of known/repaired cases and newly diagnosed cases. ^†^ These cases identified by Vscan included isolated reduced left ventricular ejection fraction (ie, less than 55% by the eyeball assessment), isolated ventricular hypertrophy and/or isolated cardiac chamber dilatation with or without reduced ventricular function suggestive for cardiomyopathies, myocarditis, or other organ damages requiring further evaluations. ^‡^ Patient had finger clubbing and decreased levels of arterial oxygen saturation.

When examined by Vscan, 32 (2.115 per 1,000 persons; CI, 1.447 to 2.985) students were identified to have isolated reduced left ventricular ejection fractions, isolated ventricular hypertrophy, and/or isolated cardiac chamber dilatation with or without reduced ventricular systolic function, suggestive of cardiomyopathies, myocarditis, or other organ damage requiring further evaluations. Re-evaluations in hospital clinics confirmed nine cases of cardiomyopathy (0.595 per 1,000 persons; 95% CI, 0.272 to 1.129): five cases of hypertrophic cardiomyopathy (0.330, per 1,000 persons; 95% CI, 0.107 to 0.771), three cases of dilated cardiomyopathy (0.198 per 1,000 persons; 95% CI, 0.040 – 0.580), and one case of arrhythmogenic right ventricular cardiomyopathy (0.070 per 1,000 persons; 95% CI, 0.001 to 0.370; Table 2).

Based on the screening protocol for the Vscan, 518 students had mitral and/or aortic valve regurgitation (due to bi-valvular involvements in some cases, the numbers of individuals are less than the number of pathologies in Table 3). After re-evaluations for identifying RHD based on the world heart federation (WHF) criteria, 143 (9.451 per 1,000 persons; 95% CI, 7.971 to 11.124) individuals were diagnosed with RHD. The rates of definite and borderline RHD based on the WHF criteria detected by standard echocardiography were 30 (1.982 per 1,000 persons; 95% CI, 1.338 to 2.829) and 113 (7.468 per 1,000 persons; 95% CI, 6.159 to 8.972), respectively. There was only one known case of definite RHD in a 16-year-old boy on a penicillin regimen.

**Table 3:** The frequency of valvular involvements among students.


	NUMBERS DETECTED BY VSCAN (%)	NUMBERS DETECTED BY STE, % (95% CONFIDENCE INTERVAL)	CRUDE RATE, PER 1000 PERSONS (95% CONFIDENCE INTERVAL)

AV pathologies			

AV regurgitation ≤mild severity	164 (1.084%)	25, 0.165% (0.107 – 0.243)	1.652 (1.070 – 2.438)

AV regurgitation >mild severity	14 (0.092%)	14, 0.092% (0.050 – 0.155)	0.925 (0.506 – 1.552)

AV billowing	10 (0.06%)	NC	…

AV prolapse of any grade	2 (0.013%)	2, 0.013% (0.001 – 0.047)	0.132 (0.016 – 0.477)

MV pathologies			

MV regurgitation ≤mild severity	863 (5.703%)	NC	…

MV regurgitation >mild severity	384 (2.538%)	90, 0.595% (0.478 – 0.730)	5.950 (4.786 – 7.307)

MV billowing	530 (3.502%)	NC	…

MV prolapse ≥mild severity	302 (1.996%)	NC	…

TV pathologies			

TV regurgitation ≤mild severity	1624 (10.733%)	NC	…

TV regurgitation ≥moderate severity	55 (0.363%)	55, 0.363% (0.274 – 0.473)	3.635 (2.740 – 4.730)

TV prolapse any grade	2 (0.013%)	2, 0.013% (0.001 – 0.047)	0.132 (0.016 – 0.477)

PV pathologies			

PV regurgitation ≤mild severity	1014 (6.701%)	NC	…

PV regurgitation ≥moderate severity	44 (0.290%)	44, 0.290% (0.211- 0.390)	2.908 (2.114 – 3.902)


AV denotes aortic valve, MV mitral valve, NC not checked, PV pulmonary valve, STE standard transthoracic echocardiography, TV tricuspid valve. * Other valvular pathologies are categorized as congenital heart disease in prior table.

Non-rheumatic VHD was calculated after excluding individuals with a diagnosis of definite RHD and those with physiologic changes (ie, valvular regurgitation ≤mild severity). Non-rheumatic VHD confirmed in hospital echocardiography clinic comprised tricuspid valve regurgitation with a minimum of moderate severity (3.635 per 1,000 persons, 95% CI, 2.740 to 4.730), any grades of tricuspid valve prolapse (0.132 per 1,000 persons; 95% CI, 0.016 to 0.477), pulmonary valve regurgitation with a minimum of moderate severity (2.908 per 1,000 persons; 95% CI, 2.114 to 3.902), mitral valve regurgitation with greater than mild severity (5.950 per 1,000 persons; 95% CI CI, 4.786 to 7.307), any grades of mitral valve prolapse (19.960 per 1,000 persons; 95% CI, 17.791 to 22.316), any grades of aortic valve regurgitation (2.578 per 1,000 persons; 95% CI, 1.834 to 3.522), and any grades of aortic valve prolapse (0.132 per 1,000 persons; 95% CI, 0.016 to 0.477). Therefore, the prevalence of individuals with non-rheumatic VHD was found to be 30.733 per 1,000 persons (95% CI, 28.041 to 33.608; Table 3).

Given the results of handheld and standard echocardiographic evaluations, the positive predictive values (PPV) for CHDs, cardiomyopathies, and >mild severity of VHDs were 88.4%, 28%, and 41%, respectively. The number of false positive findings for moderate mitral valve regurgitation was extensively higher than other heart valves. There were no false positive findings for aortic, tricuspid, and pulmonary heart valves (PPV 100% for >mild severity of aortic, tricuspid, and pulmonary heart valves). When we considered only severe mitral valve regurgitation, the PPV was found to be 100% for mitral valve too.

### Electrocardiographic findings

ECGs were taken from 15,084 students that all were interpreted by pediatric electrophysiologist. There were 166 major (11 per 1,000 persons; 95% CI, 9.4 to 12.8) and 2,734 minor (180.7 per 1,000 persons; 95% CI, 175 to 187) ECG abnormalities. The details of ECG findings are summarized in Appendix Table 4. All the 205 individuals with abnormal findings upon Vscan examinations (i.e., 172 cases with CHD, 32 cases suggestive of cardiomyopathies, and 1 pulmonary arterial hypertension) revealed 67 (32.7%) abnormal ECG findings. Of the 162 confirmed cases in hospital echocardiography clinic, 55 (34%) were found to have abnormal ECGs (Appendix Tables 5 and 6).

### Predictors of echocardiographic findings

The prevalence of SHD, CHD, and RHD were comparable between the age distributions from 6 to 18 years old, while non-rheumatic VHD was significantly increased by advancing age (Appendix Figure). After the categorization of the patients based on age [13], the frequency of CHD was significantly higher in the age group of 6 to 11 years than in the age group of 12 to 18 years (1.3% [95% CI, 1 to 1.6] vs 0.8% [95% CI, 0.6 to 1]; *P* = 0.004). All the study participants with or without CHD were compared regarding their baseline characteristics (Table 4). Those with CHD were younger (*P* = 0.026), were lighter (*P* = 0.049), and had a higher rate of parental consanguinity (*P* < 0.001) compared with their counterparts without it. Additionally, the baseline characteristics were compared across groups by the presence of SHD (Appendix Table 7), non-rheumatic VHD (Appendix Table 8), and RHD (Appendix Tables 9 and 10) with those who had normal echocardiographic screening findings.

**Table 4:** The comparison of individuals with or without CHD.


	WITH CHD N = 152	WITHOUT CHD N = 14978	P VALUE

Age, years	11.7 ± 2.9	12.2 ± 3.1	0.026

Gender			0.353

Boys	85 (55.9%)	7809 (52.1%)	

Girls	67 (44.1%)	7169 (47.9%)	

School type			0.367

Public	136 (89.5%)	13031 (87%)	

Private	16 (10.5%)	1947 (13%)	

Parental consanguinity	63 (41.4%)	3887 (26%)	<0.001

Father literacy*			0.061

High school diploma or less	122 (80.3%)	10240 (73.5%)	

University degrees	30 (19.7%)	3689 (26.5%)	

Mother literacy*			0.514

High school diploma or less	120 (78.9%)	10747 (76.7%)	

University degrees	32 (21.1%)	3265 (23.3%)	

Number of children in family			0.718

1 child	31 (20.4%)	3366 (22.5%)	

2 children	75 (49.3%)	7554 (50.4%)	

3 children	31 (20.4%)	2910 (19.4%)	

≥4 children	15 (9.9%)	1148 (7.7%)	

Height, cm	149.5 ± 16.2	151.3 ± 17.2	0.200

Weight, kg	45.3 ± 16.6	48.4 ± 18.8	0.049

Waist circumference, cm	68.8 ± 10.5	70.2 ± 11.4	0.130

Systolic blood pressure, mm Hg	113 ± 12.1	113.9 ± 12.2	0.351

Diastolic blood pressure, mm Hg	71.8 ± 7.8	72.3 ± 7.6	0.468

Arterial oxygen saturation, %	97 ± 2.6	97.2 ± 1.6	0.089


CHD denotes congenital heart disease. * These data were not available in 6–7% of individuals. Data are presented as number (%) or mean ± SD.

**Figure 2:**
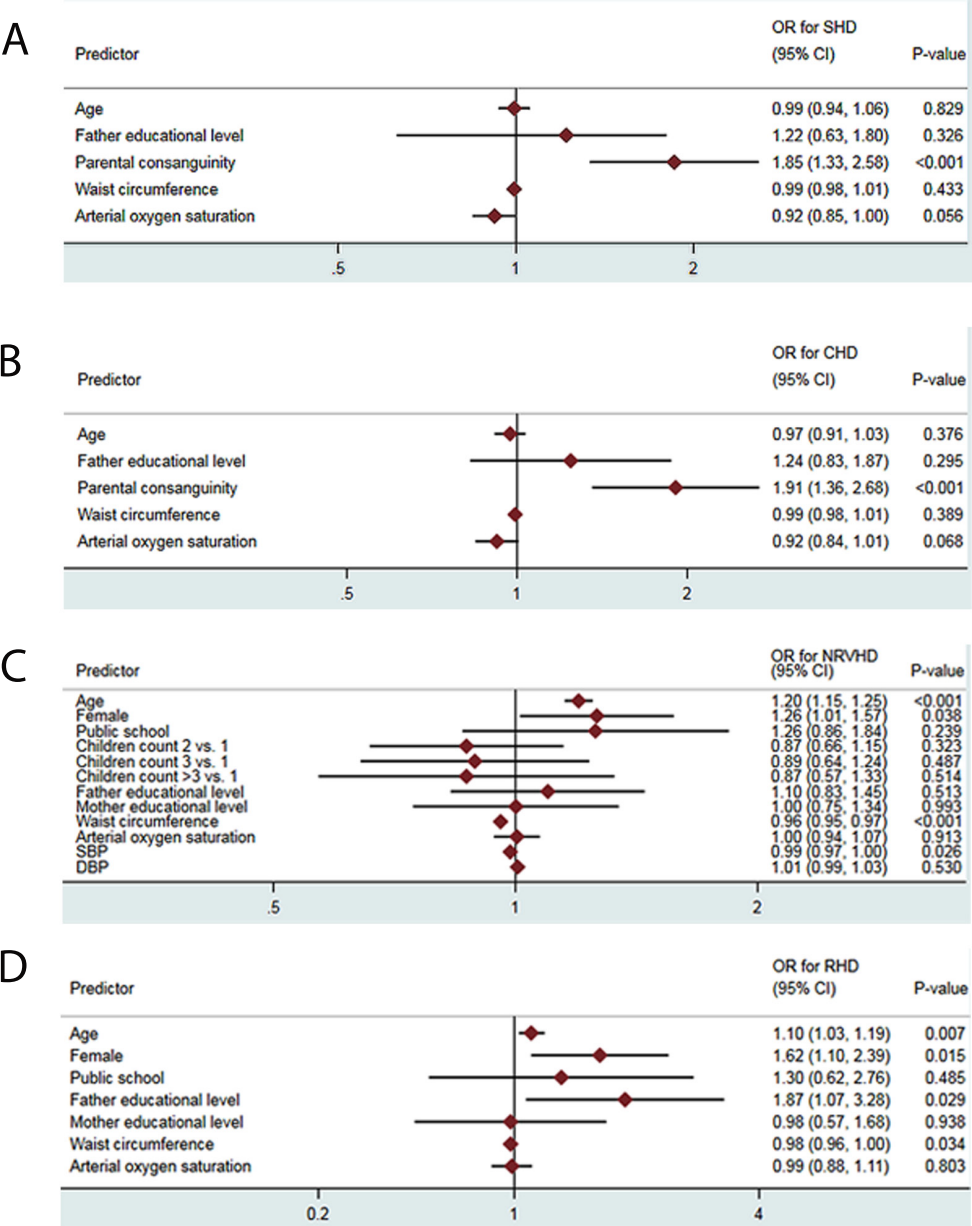
Multivariable analyses showing predictors of echocardiographic findings among individuals with **(A)** SHD, **(B)** CHD, **(C)** non-rheumatic VHD, and **(D)** RHD. The horizontal lines indicate the 95% confidence intervals (CIs) for the odds ratios (ORs), and the diamonds in the middle of the horizontal lines indicate the OR of the predictor entered in the multivariable model.

After the variables were entered into multivariable models, parental consanguinity was the strongest predictor of CHD and SHD (odds ratio [OR], 1.907, 95% CI, 1.358 to 2.680; *P* < 0.001 and OR, 1.855, 95% CI, 1.334 to 2.579; *P* < 0.001, respectively). Apropos the predictors of valvular pathologies, the strongest predictors of non-rheumatic VHD and RHD were the female sex (OR, 1.262, 95% CI, 1.013 to 1.573; *P* = 0.038) and a lower level of fathers’ literacy (OR, 1.872, 95% CI, 1.068 to 3.281; *P* = 0.029), respectively. The findings of the multivariable analyses are summarized in Figure 2.

### Therapeutic interventions

Of SHD pathologies found among the study population, three students with atrial septal defect underwent surgical or interventional treatments and students with definite RHD received medical therapies. Other pathologies, including both CHD and VHD, were scheduled to be followed up or to receive medical therapies if they had mild to moderate symptoms.

## Discussion

In this large-scale study we report the implementation of echocardiographic screening for the detection of SHD among a school-age population. The latest Global Burden of Disease (GBD) study reported the prevalence rates of people living with CHD increased by 18.7% from 1990 to 2017 [14]. In a report from the United States, the frequency of CHD was 15.57 per 1,000 persons for those aged between 6 and 12 years and 12.62 per 1,000 persons for those aged between 13 and 17 years [15]. Marelli et al. [16] showed that the prevalence of CHD among Canadian children was 13.11 per 1,000 persons. In a study on 11,168 soccer players (age 15–17 y, 95% male), 175 CHD cases (15.7 per 1,000 persons) were identified [17]. Given these findings, the prevalence of CHD in our study is relatively close to that of the GBD study and is lower than the rates reported by the mentioned studies. Nevertheless, prevalence has been influenced by the methods of screening, population selection in different studies, and the socioeconomic index of population. The prenatal screening of CHD is not routinely implemented in Iran, and echocardiographic screening during pregnancy is performed in some individuals; consequently, an echocardiographic screening protocol during peripartum, preschool, and/or mid-school periods in any individual might be recommended.

Cardiomyopathy is a life-threatening phenomenon found in 0.07% of athletes (age ≤ 35 y) at pre-participation screening [18]. Overall, the identification of cardiomyopathy requires follow-ups and further evaluations. It has been demonstrated that the timing and severity of presentation in children with cardiomyopathy are related to the type of cardiomyopathy, as well as to the genetic and ethnic factors [5]. In a prospective study, the majority of sudden cardiac deaths caused by cardiomyopathies were developed in individuals with normal echocardiographic screening at participation time [17]. We also found cardiomyopathies in 0.065% of the children recruited in our investigation; however, all the aforementioned factors should be taken into account for the detection of cardiomyopathy in any echocardiographic screening protocols.

Marijon et al. [19] showed a prevalence of 21.5 per 1,000 and 30.4 per 1,000 persons for RHD in Cambodia and Mozambique, respectively. Rémond et al. [20] also demonstrated that children with borderline RHD were more likely to develop definite RHD compared with those with other minor nonspecific valvular abnormalities. Given these notions and the lack of further data on long-term follow-ups, it might be logical to consider only definite RHD as the burden of RHD so as to avoid overestimation. On the other hand, close follow-ups of young children with borderline RHD can be the cornerstone of any programs aimed at eradicating RHD. We found an increased risk of RHD among female children, older children, slimmer children, and children with fathers’ literacy levels of less than university qualifications. Both former factors have been found in prior studies [21], but both latter factors are indicators of low socioeconomic status [8]. It is worth noting that due to high economic levels in the Tehran urban areas by comparison with regions with limited resources, our findings with regard to RHD cannot be generalizable to the entire Iranian population.

Moderate or severe non-rheumatic VHD was found in 3.7% of an adult population using a systematic echocardiographic screening [22]. Among 16-year-old individuals, the prevalence of aortic valve regurgitation and mitral valve prolapse was 0.5%, while no one was diagnosed with mitral valve regurgitation or tricuspid and pulmonary valve insufficiencies [17]. The prevalence of non-rheumatic VHD has not been systematically explored among children (5–19 y), which explains the dearth of data on children in the GBD study [23]. We think that this missing information may be explained by the notion that the majority of non-rheumatic VHD cases among children are non-pathologic and their impact on public health is less than that of VHD among adults. On the other hand, borderline RHD is a pathologic valvular regurgitation and can also be considered non-rheumatic VHD; nevertheless, such cases need to be followed up by echocardiographic evaluations with regard to the possibility of the development of RHD [24].

With respect to pathologies, only some of students with CHD and RHD had already been diagnosed, while some cases required close follow-ups using physical examination and/or echocardiographic evaluations due to the progressive nature of pathologies and their hemodynamic consequences. Given the progressive nature of SHDs found in our study, the early diagnosis can help us to decrease morbidities and life-threatening consequences which can also enhance the cost of care (e.g. pulmonary artery hypertension or risk of arrhythmia following missed atrial septal defect or sudden cardiac death due to cardiomyopathies). Given the good to excellent agreement between handheld machine and standard echocardiography in the majority of significant lesions [11], the current investigation provides valuable data on the magnitude of the SHD burden in the young population and the paramount role of echocardiographic screening for identifying structural pathologies irrespective of individuals’ symptoms or prior histories. Therefore, the screening of SHD at school-age can be of utmost importance for low- and middle-income countries to detect undiagnosed SHDs among population.

Several factors predispose individuals to a higher risk of disease development, particularly pathologies with a genetic background [25]. Some studies have emphasized the role of genes in the causation of CHD and cardiomyopathy [5,26], and recommended against inbreeding and consanguineous marriage, particularly in developing countries [26]. Surprisingly, we also found that children with CHD were more likely to have parental consanguinity than those without it. In addition, ECG findings can also be part of a screening protocol since it has been found to improve the sensitivity of pre-participation cardiovascular screening in athletes [27]. In our study, more than half of the patients with CHD had abnormal ECGs; the abnormalities were, however, unspecific. It should be taken into account that ECG examinations can promote further false-positive findings if they are incorporated into screening protocols.

Future study should consider some important issues. First, the screening tool must apply a standard echocardiographic evaluation. Second, after providing more funding supports, it would be of utmost importance to evaluate the comparison between handheld machine and standard echocardiography among all students, with or without any findings. Finally, it has been demonstrated that the majority of cardiomyopathies cannot be detected by echocardiographic screening among adolescents and follow-up is required to distinguish all asymptomatic cases [17], therefore the long-term follow-up of all participants with or without abnormal findings is mandatory.

### Study limitations

In the interpretation of the results of the present study, the following limitations should be taken into consideration. The main shortcoming is the lack of the continuous wave and pulsed wave Doppler modalities in Vscan preventing from more definite measurements. Be that as it may, it is deserving of note that we conducted a study on the accuracy of a pocket-sized ultrasound device for the screening of SHD and found that the agreement between the standard and pocket-sized echocardiographic machines was good or excellent for SHD among children with high sensitivity and specificity in hospital-based and school-based evaluations [11]. The prevalence rate of patent foramen ovale in our investigation is surely less than the true prevalence rate since its detection requires contrast echocardiography and transesophageal echocardiography [28]. Another note is that the rate of ventricular septal defect must have also been underestimated, which can be explained by the notion that the majority of such defects (up to 75%) close spontaneously by 120 months [29]. In addition, we found lower rates of bicuspid aortic valve (BAV) in our population compared to other studies. Malhotra et al. [17] found BAV in 0.6% of population (11,168 soccer players aged 15–17 y, 95% male) and Basso et al. [30] also found BAV in 0.5% of small school-based population in Italy (817 apparently healthy 10-year olds). Movahed et al. [31] also found 0.5% prevalence of BAV in a small population of athletic database (1,172 individuals, 67% male). It has been shown that the sensitivity of transthoracic echocardiography is approximately 60% for detecting BAV when non-expert investigators performed echocardiography [32]. Therefore, given the use of handheld echocardiography in our study, the lower rates of BAV can be explained by lower accuracy of devices used in our study.

## Conclusions

The present study provides evidence regarding the relatively high prevalence of SHD based on handheld echocardiographic screening among a young group of subjects irrespective of their symptoms or prior histories. The majority of the pathologies were newly diagnosed in the studied population, underscoring the paramount role of echocardiographic screening among school-age children. Our findings merit further population-based studies to explore the probable utility of echocardiographic screening for identifying SHD and its potential to improve care delivery in communities. Further studies are required to delineate the economic aspects of handheld echocardiography for community-based screening.

## Additional File

The Additional file for this article can be found as follows:

10.5334/gh.1121.s1 Supplementary materials. Further details for the study methods, including protocols, data collection, and data interpretation.
