# Supplementary material for: Prevalence of Structural Heart Diseases Detected by Handheld Echocardiographic Device in School-Age Children in Iran: The SHED LIGHT Study
Source: Glob Heart. 2022 Jun 15;17(1):39. doi: 10.5334/gh.1121 (PMC9205369; doi:10.5334/gh.1121)
Supplement: Supplementary materials. — Further details for the study methods, including protocols, data collection, and data interpretation. [file gh-17-1-1121-s1.pdf]

Prevalence of structural heart diseases detected by handheld echocardiographic device in school-age children in Iran: The SHED LIGHT study

Supplementary materials

Supplementary methods- Study protocol and population selection

Supplementary methods- Echocardiographic examination

Supplementary methods- Electrocardiographic examinations

Supplementary table S1- Diagnostic criteria for screening of RHD using Vscan and standard transthoracic echocardiography

Supplementary table S2- The frequency of newly diagnosed CHD and cardiomyopathies

Supplementary table S3- The frequency of individuals with concomitant CHD

Supplementary table S4- ECG findings among all individuals

Supplementary table S5- ECG findings in patients with SHD upon Vscan examinations and corresponding diagnosis upon standard echocardiographic examinations

Supplementary table S6- The details of echocardiographic findings upon standard echocardiographic examinations and corresponding ECG diagnosis

Supplementary table S7- The comparison of individuals with or without total SHD

Supplementary table S8- The comparison of individuals with or without non-rheumatic VHD

Supplementary table S9- The comparison of individuals with or without RHD

Supplementary table S10- The comparison of individuals with borderline RHD, definite RHD, and without RHD

Supplementary Fig. S1 The areas of study located in the Tehran urban territory

Supplementary Fig. S2 Age distribution of the studied population

Supplementary Fig. S3 The proportion of students with (A) CHD, (B) non-rheumatic VHD, (C) RHD, and (D) SHD in age groups from 6 to 18 years old.

References

Supplementary methods

Study protocol and population selection

The present study was conducted in the Tehran urban area, Iran. At the commencement of the study, the population of Tehran, divided into 22 municipality districts, was more than 8.9 million. The total number of schools and/or educational centers in Tehran urban area was around 3900, and the total population of school-age children was approximately 1.2 million at study commencement. The study was performed between October 2017 and December 2018 in selected schools of Tehran. The sample size from each region was proportionate to the population of the district. Given the probability of absence in examinations and parental non-consent for examination, the sample size was aimed to reach approximately 15 000 students for the final cohort. Random school selection was done by considering an equal number of single-sex schools for boys and girls. The number of private schools was obtained based on the proportion of the students studying at private schools confirmed by the Tehran Department of Education. After the random selection of schools, the members of the schools' management departments were informed regarding the details of the study, and they were requested to transfer the details of the study protocol to all the parents and students via group meetings in school and/or telephone interviews. All the parents provided consent at least 14 days before the examinations.

Supplementary methods

Echocardiographic examinations

Echocardiographic examinations were performed using a pocket-sized ultrasound device (Vscan, GE Healthcare, Milwaukee, WI, USA). The Vscan echo machine weighs 391 g and is 13.5 × 7.4 × 2.8 cm in size. The device provides 2D grayscale and color Doppler images obtained with a 1.7–3.8 MHz phased-array transducer. It does not possess zoom, spectral Doppler, or the ability to perform velocity or time measurements.

The interpretation of valvular heart disease (VHD) was qualitatively performed. Valvular regurgitation was categorized into mild, moderate, or severe based on color-flow mapping. For the assessment of the severity of aortic insufficiency and pulmonary insufficiency, eyeball assessment of the regurgitant jet width was drawn upon. Accordingly, regurgitant jets of less than 20%, between 20% and 50%, and greater than 50% of the annulus diameter were considered mild, moderate, and severe regurgitation, respectively. Mitral regurgitation and tricuspid regurgitation were estimated based on the presence of central or lateral jets and by the eyeball assessment of the jet area to the left atrium and the right atrium, respectively. The grading of severity was similar to that in the aortic and tricuspid valves. The prolapse of the valves and valvular stenosis were interpreted as absent or present for being pathologic even for a mild lesion. Valvular leaflet motion, identified as normal or any restrictions or malcoaptation, was assessed by color turbulency. The identification of rheumatic heart disease (RHD) was based on the criteria of the World Heart Federation (WHF) [1]. However, since we used pocket-sized echocardiography with on-site interpretation, the simplified version was used for the detection of

RHD (Table S1) [2]. The measurement of ventricular systolic function was performed visually (ie, normal function vs mild, moderate, and severe dysfunction). The eyeball evaluation of the left ventricular ejection fraction was classified into 4 groups of greater than 55%, between 55% and 45%, between 45% and 35%, and less than 35% as normal, mild, moderate, and severe dysfunction, respectively. The heart chambers were evaluated qualitatively as normal, dilated, or hypoplastic/small. Large vessel abnormalities were also qualitatively reported as normal, dilated, or stenotic based on 2D images and color study. Any other congenital heart disease (CHD) or anatomic variations were reported when detected.

Supplementary methods

Electrocardiographic examinations

The electrocardiogram (ECG) was recorded by 4 technicians with more than 5 years of experience. The technicians work in a tertiary hospital and take at least 50 ECGs per working day. At the time of the examination, all ECGs were checked by cardiologists. In the event significant noises rendered an ECG uninterpretable, another ECG was recorded. The interpretation of traces was performed according to current international standards by a pediatric cardiologist with a fellowship in electrophysiology [3-7].

Supplementary table S1- Diagnostic criteria for screening of RHD using Vscan and standard transthoracic echocardiography

**Screening criteria for Vscan**	
Pathologic MV regurgitation	□ MV regurgitation jet >2 cm presents in any views which was estimated based on the scale at the border of sector and/or the eyeball assessment of jet area to the left atrium area >20%<br>□ Both conditions in the absence of concomitant prolapse and congenital anomalies
Pathologic AV regurgitation	□ AV regurgitation presents in any views without prolapse and congenital anomalies
**Screening criteria for standard transthoracic echocardiography using WHF**	
Pathologic MV regurgitation	All of: 1) Regurgitation jet seen in 2 views; 2) with at least one view >2 cm; 3) with high velocity (>3 m/s); and 4) pan systolic jet in at least one envelope
Pathologic AV regurgitation	All of: 1) Regurgitation jet seen in 2 views; 2) with at least one view >1 cm; 3) with high velocity (>3 m/s) in early diastole; and 4) pan-diastolic jet in at least one envelope
Morphological changes of MV	Two or more of: 1) anterior MV leaflet thickening ≥3 mm; 2) chordal thickening; 3) restricted leaflet motion; 4) and excessive leaflet tip motion during systole
Morphological changes of AV	Two or more of: 1) irregular or focal thickening of AV; 2) coaptation defect; 3) restricted leaflet motion; and 4) prolapse
Definite RHD	□ Definite A: Pathological MV regurgitation plus morphological changes of the mitral valve (i.e. two or more)<br>□ Definite B: Mitral stenosis .4 mmHg<br>□ Definite C: Pathological AV regurgitation plus morphological changes of the aortic valve (i.e. two or more)<br>□ Definite D: Borderline disease of both MV and AV
Borderline RHD	□ Borderline A: Morphological features of the MV (i.e. at least two) without pathological MV regurgitation or mitral stenosis<br>□ Borderline B: Pathological MV regurgitation<br>□ Borderline C: Pathological AV regurgitation

AV denotes aortic valve, MV mitral valve, RHD rheumatic heart disease, and WHF world heart federation

Supplementary table S2- The frequency of newly diagnosed CHD and cardiomyopathies

	**Findings, number (%)**	**Crude rate, per 1000 persons**	**95% confidence interval**
Atrial septal defect	35 (0.23%)	2.31	1.6 – 3.2
Patent foramen ovale	33 (0.22%)	2.18	1.5 – 3
Bicuspid aortic valve	18 (0.13%)	1.19	0.8 – 2
Patent ductus arteriosus	16 (0.10%)	1.06	0.6 – 1.7
Ventricular septal defect	9 (0.06%)	1.06	0.6 – 1.7
Cardiomyopathies	9 (0.06%)	0.6	0.2 – 1.1
Sub-aortic web	4 (0.026%)	0.26	0.07 – 0.7
Persistent LSVC	3 (0.019%)	0.19	0.04 – 0.6
Coarctation of aorta	2 (0.013%)	0.13	0.01 – 0.5
Isolated PA dilatation	2 (0.013%)	0.13	0.01 – 0.5
Isolated sinus valsalva dilatation	2 (0.013%)	0.13	0.01 – 0.5
Isolated right aortic arch	1 (0.007%)	0.07	0.002 – 0.3
Supravalvular mitral mass	1 (0.007%)	0.07	0.002 – 0.3
Quadricuspid aortic valve	1 (0.007%)	0.07	0.002 – 0.3
Isolated aorta dilatation	1 (0.007%)	0.07	0.002 – 0.3
Situs inversus	1 (0.007%)	0.07	0.002 – 0.3

CHD denotes congenital heart disease, LSVC left superior vena cava, PA pulmonary artery, and STE standard transthoracic echocardiography

Supplementary table S3- The frequency of individuals with concomitant CHD

	**Number (%)**	**95% confidence interval**
Concomitant PFO and PDA	2 (0.013%)	0.01 – 0.5
Concomitant PFO and ASD	2 (0.013%)	0.01 – 0.5
Concomitant CoA and BAV	2 (0.013%)	0.01 – 0.5
Concomitant PFO and VSD	1 (0.007%)	0.002 – 0.3
Concomitant ASD and VSD	1 (0.007%)	0.002 – 0.3
Concomitant VSD and BAV	1 (0.007%)	0.002 – 0.3
Concomitant right aortic arch and situs inversus	1 (0.007%)	0.002 – 0.3

ASD denotes atrial septal defect, BAV bicuspid aortic valve, CoA coarctation of aorta, PDA patent ductus arteriosus, PFO patent foramen ovale, and VSD ventricular septal defect

Supplementary table S4- ECG findings among all individuals

Major and minor electrocardiographic findings in studied population

	**Frequency (%)**	**Crude rate, per 1000 persons**	**95% confidence interval**
**Major findings**			
PAC	50 (0.33%)	3.31	2.46 – 4.36
PVC	44 (0.29%)	2.91	2.12 – 3.91
QT interval >470 ms	22 (0.15%)	1.46	0.91 – 2.20
Ventricular pre-excitation	13 (0.086%)	0.86	0.45 – 1.47
Complete RBBB	10 (0.066%)	0.66	0.31 – 1.21
PR interval >200 ms	9 (0.059%)	0.6	0.27 – 1.13
Non-conducted PAC	8 (0.053%)	0.53	0.22 – 1.04
SND*	6 (0.04%)	0.40	0.14 – 0.86
LPFB	4 (0.026%)	0.26	0.07 – 0.67
Paced rhythm**	2 (0.013%)	0.13	0.01 – 0.47
Epsilon wave	1 (0.007%)	0.07	0.002 – 0.36
**Minor findings**			
Incomplete RBBB or RVCD	1071 (7.1%)	71	66.95 – 75.21
Sinus arrhythmia	887 (5.9%)	58.8	52.10 – 62.67
Sinus tachycardia	562 (3.7%)	37.25	34.29 – 40.40
Sinus bradycardia	162 (1%)	10.74	9.15 – 12.51
Early repolarization	52 (0.34%)	3.45	2.57 – 4.51
Sinus pause	44 (0.29%)	2.91	2.12 – 3.91
Pseudo delta wave	35 (0.22%)	2.32	1.61 – 3.22
ST segment changes	29 (0.19%)	1.92	1.28 – 2.76
Atrial rhythm	28 (0.18%)	1.86	1.23 – 2.68
Tall T wave	19 (0.12%)	1.26	0.75 – 1.96
Minimal ventricular pre-excitation	17 (0.11%)	1.13	0.65 – 1.80
Q wave	3 (0.02%)	0.2	0.04 – 0.58

* Suspicious for sinus node dysfunction requiring further evaluations

** Patients with paced rhythm had cardiac block and sinus node dysfunction causing bradyarrhythmia which were counted once as mentioned diagnoses in this table

Abbreviations: PAC = premature atrial contraction; PVC = premature ventricular contraction; ms = millisecond; RBBB = right bundle branch block; SND = sinus node dysfunction; LPFB = left posterior fascicular block; RVCD = right ventricular contraction delay

Supplementary table S5- ECG findings in patients with SHD upon Vscan examinations and corresponding diagnosis upon standard echocardiographic examinations

	**Frequency of ECG findings in individuals with Vscan abnormalities*** **(n = 205)**	**CHD and CMP upon Vscan examinations found as normal on STE (n = 43)**	**CHD and CMP upon STE examinations[†] (n = 162)**
ECG findings			
**Normal**	**138 (67.3%)**	**31 (72.1%)**	**107 (66.1%)**
**Abnormal**	**67 (32.7%)**	**12 (27.9%)**	**55 (33.9%)**
Sinus tachycardia	28 (41.8%)	4 (33.3%)	24 (43.6%)
Incomplete RBBB	16 (23.9%)	3 (25%)	13 (23.6%)
Sinus arrhythmia	6 (8.9%)	1 (8.3%)	5 (9.1%)
RVCD	4 (6%)	1 (8.3%)	3 (5.5%)
Complete RBBB	3 (4.5%)	0	3 (5.5%)
Atrial rhythm	3 (4.5%)	1 (8.3%)	2 (3.6%)
Early repolarization	2 (3%)	1 (8.3%)	1 (1.8%)
Sinus bradycardia	1 (1.5%)	0	1 (1.8%)
PVC	1 (1.5%)	0	1 (1.8%)
Sinus pause	1 (1.5%)	1 (8.3%)	0
Epsilon wave	1 (1.5%)	0	1 (1.8%)
Tall T wave	1 (1.5%)	0	1 (1.8%)

* Pathologies include CHDs (n = 172) listed in table 2, CMPs (n = 32), and pulmonary arterial hypertension (n = 1)

† Pathologies include CHDs (n = 152) listed in table 2, CMPs (n = 9), and pulmonary arterial hypertension (n = 1)

Supplementary table S6- The details of echocardiographic findings upon standard echocardiographic examinations and corresponding ECG diagnosis

ECG findings	CHD and CMP findings upon STE examination (n = 55)
Sinus tachycardia	8 ASD, 7 PFO, 3 BAV, 1 Repaired TGA, 1 Repaired CoA, 1 CoA, 1 PDA, 1 PAH, 1 CMP
Incomplete RBBB	5 ASD, 3 VSD, 2 ASD/PFO, 1 PFO, 1 VSD/BAV, 1 PDA
Sinus arrhythmia	3 ASD, 1 BAV, 1 VSD
RVCD	1 Right aortic arch, 1 ASD, 1 CMP
Complete RBBB	1 TFTC, 1 PS, 1 VSD
Atrial rhythm	1 CMP, 1 PDA
Early repolarization	1 BAV
Sinus bradycardia	1 ASD
PVC	1 ASD
Epsilon wave	1 ARVC
Tall T wave	1 CMP

ASD denotes atrial septal defect, BAV bicuspid aortic valve, CHD congenital heart disease, CMP cardiomyopathy, CoA coarctation of aorta, ECG electrocardiogram, PAH pulmonary arterial hypertension, PDA patent ductus arteriosus, PFO patent foramen ovale, PS pulmonary valve stenosis, PVC premature ventricular contraction, RBBB right bundle branch block, RVCD right ventricular conduction delay, SHD structural heart disease, STE standard transthoracic echocardiography, TFTC tetralogy of Fallot total correction, TGA transposition of great artery, VSD ventricular septal defect

Supplementary table S7- The comparison of individuals with or without total SHD

	With SHD* (n = 162)	Without SHD (n = 14968)	P value
Age, years	11.9 ± 2.9	12.2 ± 3.1	0.162
Gender			0.386
Boys	90 (55.6%)	7804 (52.1%)	
Girls	72 (44.4%)	7164 (47.9%)	
School type			0.345
Public	145 (89.5%)	13022 (87%)	
Private	17 (10.5%)	1946 (13%)	
Parental consanguinity	66 (40.7%)	3888 (26%)	<0.001
Father literacy†			0.079
High school diploma or less	129 (79.6%)	10233 (73.5%)	
University degrees	33 (20.4%)	3686 (26.5%)	
Mother literacy†			0.612
High school diploma or less	127 (78.4%)	10740 (76.7%)	
University degrees	35 (21.6%)	3262 (23.3%)	
Number of children in family			0.870
1 child	37 (22.8%)	3360 (22.4%)	
2 children	78 (49.9%)	7551 (50.4%)	
3 children	32 (19.8%)	2909 (19.4%)	
≥4 children	15 (9.3%)	1148 (7.7%)	
Height, cm	150.4 ± 16.3	151.34 ± 17.1	0.508
Weight, kg	46.1 ± 16.6	48.4 ± 18.8	0.126
Body mass index, kg/m^2	19.8 ± 4.5	20.4 ± 4.9	0.135
Waist circumference, cm	69.2 ± 10.5	70.2 ± 11.4	0.296
Systolic blood pressure, mm Hg	113.6 ± 12.4	113.9 ± 12.2	0.763
Diastolic blood pressure, mm Hg	72.1 ± 7.8	72.3 ± 7.6	0.705
Arterial oxygen saturation, %	97 ± 2.5	97.2 ± 1.5	0.095

SHD denotes structural heart disease

* Pathologies include CHDs (n = 152) listed in table 2, CMPs (n = 9), and pulmonary arterial hypertension (n = 1)

† These data were not available in about 7% of individuals

Data are presented as number (%) or mean ± SD

Supplementary table S8- The comparison of individuals with or without non-rheumatic VHD

	With non-rheumatic VHD (n = 465)	Without non-rheumatic VHD (n = 14665)	P value
Age, years	13.2 ± 3.1	12.2 ± 3	<0.001
Gender			<0.001
Boys	187 (40.2%)	7707 (52.6%)	
Girls	287 (59.8%)	6958 (47.4%)	
School type			0.015
Public	422 (90.8%)	12745 (86.9%)	
Private	43 (9.2%)	1920 (13.1%)	
Parental consanguinity	129 (27.7%)	3825 (26.1%)	0.423
Father literacy*			0.035
High school diploma or less	346 (77.9%)	10016 (73.4%)	
University degrees	98 (22.1%)	3621 (26.6%)	
Mother literacy*			0.045
High school diploma or less	359 (80.7%)	10508 (76.6%)	
University degrees	86 (19.3%)	3211 (23.4%)	
Number of children in family			0.040
1 child	93 (20%)	3304 (22.5%)	
2 children	220 (48.3%)	7409 (50.5%)	
3 children	105 (22.6%)	2836 (19.3%)	
≥4 children	47 (10.1%)	1116 (7.6%)	
Height, cm	155.3 ± 17.1	151.2 ± 17.1	<0.001
Weight, kg	47.7 ± 17	48.4 ± 18.8	0.415
Body mass index, kg/m^2	19.2 ± 4.3	20.4 ± 4.9	<0.001
Waist circumference, cm	67.7 ± 10	70.3 ± 11.4	<0.001
Systolic blood pressure, mm Hg	111.7 ± 12	114 ± 12.2	<0.001
Diastolic blood pressure, mm Hg	71.6 ± 7.8	72.3 ± 7.6	0.070
Arterial oxygen saturation, %	97.4 ± 1.8	97.2 ± 1.6	0.003

VHD denotes valvular heart disease

* These data were not available in about 7% of individuals

Data are presented as number (%) or mean ± SD

Supplementary table S9- The comparison of individuals with or without RHD

	With RHD (n = 143)	Without RHD (n = 14987)	P value
Age, years	13.1 ± 3.1	12.2 ± 3	<0.001
Gender			<0.001
Boys	53 (37.1%)	7841 (52.3%)	
Girls	90 (62.9%)	7146 (47.7%)	
School type			0.059
Public	132 (92.3%)	13035 (87%)	
Private	11 (7.7%)	1952 (13%)	
Parental consanguinity	36 (25.2%)	3918 (26.1%)	0.793
Father literacy*			0.001
High school diploma or less	118 (86.1%)	10244 (73.5%)	
University degrees	19 (13.9%)	3700 (26.5%)	
Mother literacy*			0.065
High school diploma or less	115 (83.3%)	10752 (76.7%)	
University degrees	23 (16.7%)	3274 (23.3%)	
Number of children in family			0.363
1 child	24 (16.8%)	3373 (22.5%)	
2 children	75 (52.4%)	7554 (50.4%)	
3 children	30 (21%)	2911 (19.4%)	
≥4 children	14 (9.8%)	1149 (7.7%)	
Height, cm	155 ± 16.8	151.3 ± 17.1	0.011
Weight, kg	49.4 ± 18.2	48.4 ± 18.8	0.521
Body mass index, kg/m^2	19.9 ± 4.6	20.3 ± 4.9	0.219
Waist circumference, cm	69.1 ± 10.7	70.2 ± 11.4	0.270
Systolic blood pressure, mm Hg	113.3 ± 12.7	113.9 ± 12.2	0.535
Diastolic blood pressure, mm Hg	71.9 ± 7.8	72.3 ± 7.6	0.536
Arterial oxygen saturation, %	97.4 ± 1.8	97.2 ± 1.6	0.186

RHD denotes rheumatic heart disease

* These data were not available in about 7% of individuals

Data are presented as number (%) or mean ± SD

Supplementary table S10- The comparison of individuals with borderline RHD, definite RHD, and without RHD

	Definite RHD (n = 30)	Borderline RHD (n = 113)	Without RHD (n = 14987)	P value
Age, years	13.4 ± 3	13 ± 3.2	12.2 ± 3	0.005
Gender				<0.001
Boys	12 (40%)	41 (36.3%)	7841 (52.3%)	
Girls	18 (60%)	72 (63.7%)	7146 (47.7%)	
School type				0.165
Public	28 (93.3%)	104 (92%)	13035 (87%)	
Private	2 (6.7%)	9 (8%)	1952 (13%)	
Parental consanguinity	7 (23.3%)	29 (25.7%)	3918 (26.1%)	0.935
Father literacy*				0.003
High school diploma or less	24 (82.8%)	94 (87%)	10244 (73.5%)	
University degrees	5 (17.2%)	14 (13%)	3700 (26.5%)	
Mother literacy*				0.167
High school diploma or less	25 (86.2%)	90 (82.6%)	10752 (76.7%)	
University degrees	4 (13.8%)	19 (17.4%)	3274 (23.3%)	
Number of children in family				0.135
1 child	8 (26.7%)	16 (14.2%)	3373 (22.5%)	
2 children	18 (60%)	57 (50.4%)	7554 (50.4%)	
3 children	2 (6.7%)	28 (24.8%)	2911 (19.4%)	
≥4 children	2 (6.7%)	12 (10.6%)	1149 (7.7%)	
Height, cm	155.6 ± 15.7	154.8 ± 17.1	151.3 ± 17.1	0.038
Weight, kg	49.3 ± 18.5	49.5 ± 18.3	48.4 ± 18.8	0.813
Body mass index, kg/m^2	19.6 ± 4.5	19.9 ± 4.6	20.3 ± 4.9	0.453
Waist circumference, cm	69.3 ± 10.7	69.1 ± 10.7	70.2 ± 11.4	0.542
Systolic blood pressure, mm Hg	116.4 ± 13.4	112.4 ± 12.4	113.9 ± 12.2	0.233
Diastolic blood pressure, mm Hg	72.7 ± 8	71.7 ± 7.7	72.3 ± 7.6	0.662
Arterial oxygen saturation, %	97.7 ± 1.4	97.3 ± 1.9	97.2 ± 1.6	0.234

RHD denotes rheumatic heart disease

* These data were not available in about 7% of individuals

Data are presented as number (%) or mean ± SD

Supplementary Fig S1. The areas of study located in the Tehran urban territory

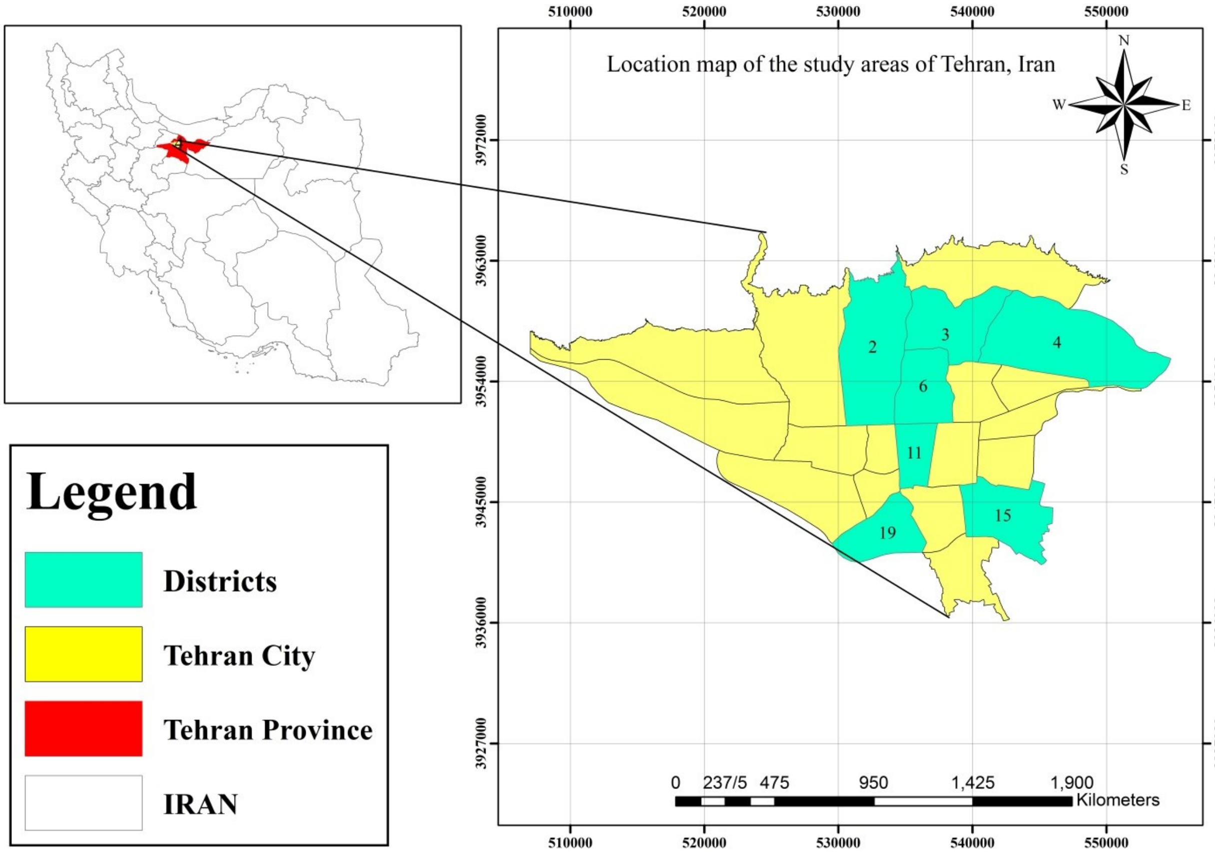

Supplementary Fig. S2 Age distribution of the studied population

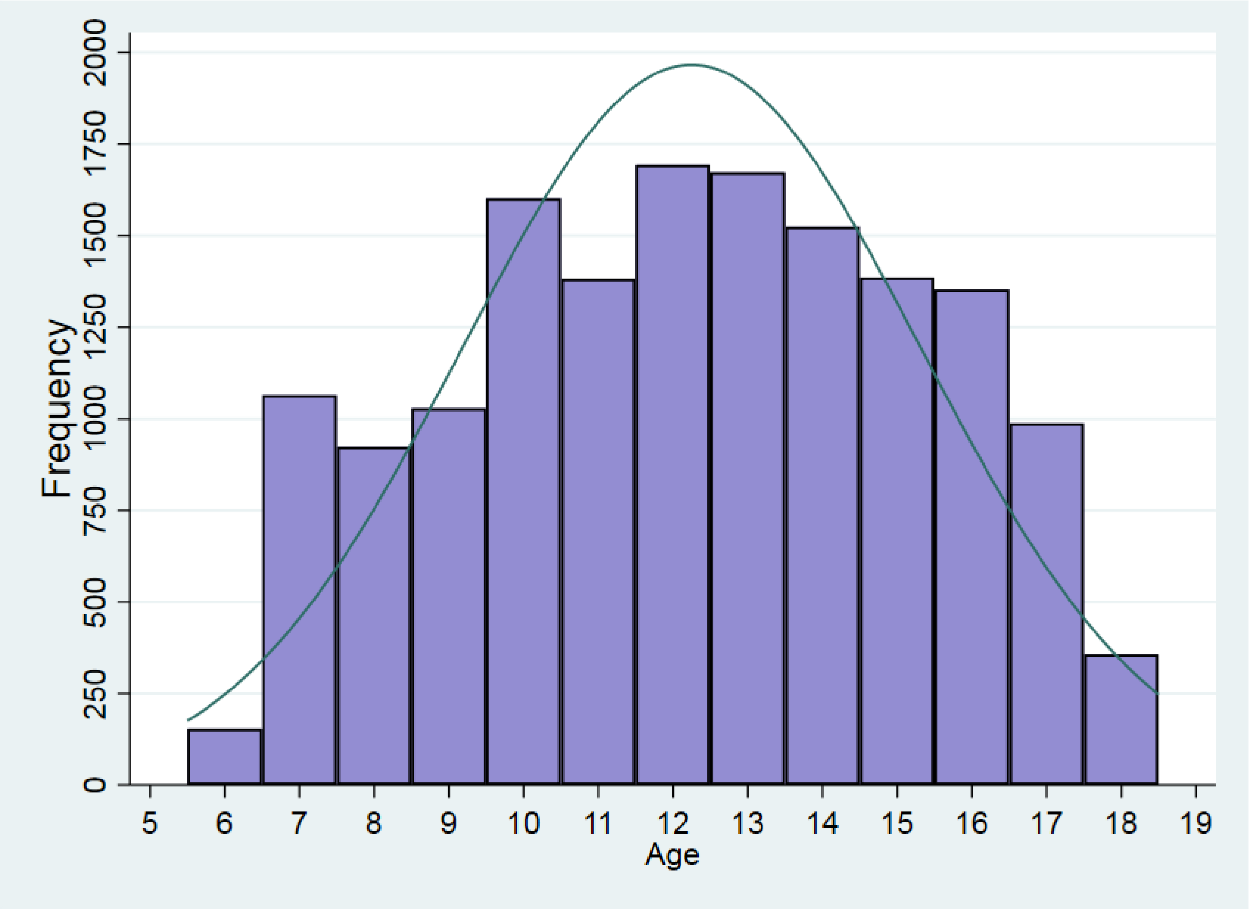

Supplementary Fig. S3 The proportion of students with (A) CHD, (B) non-rheumatic VHD, (C) RHD, and (D) SHD in age groups from 6 to 18 years old.

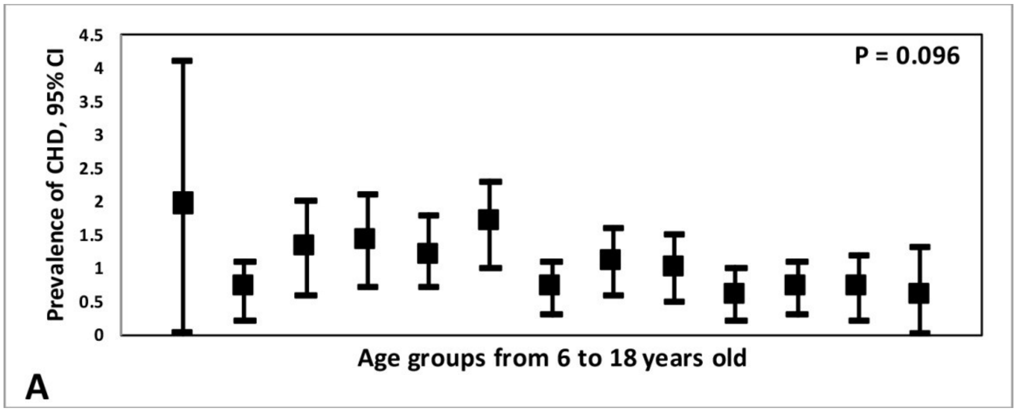

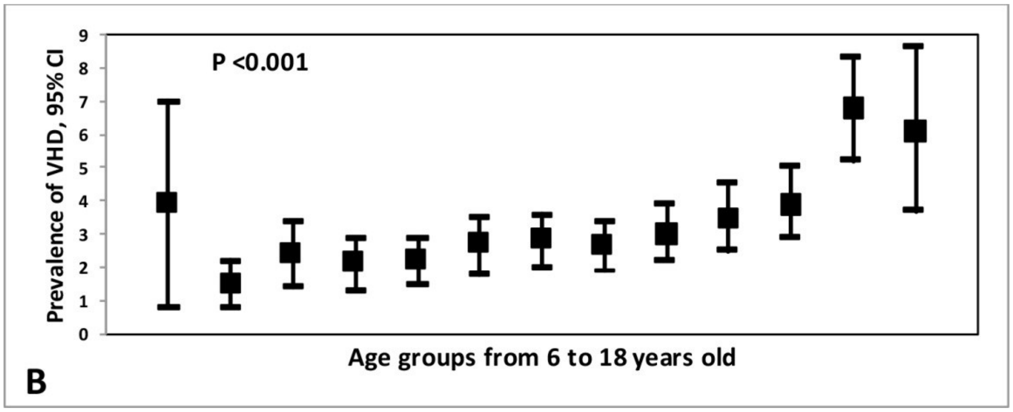

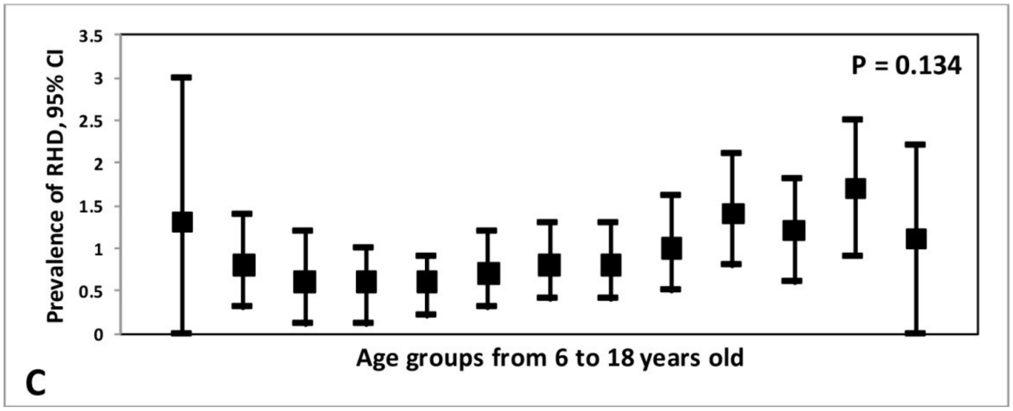

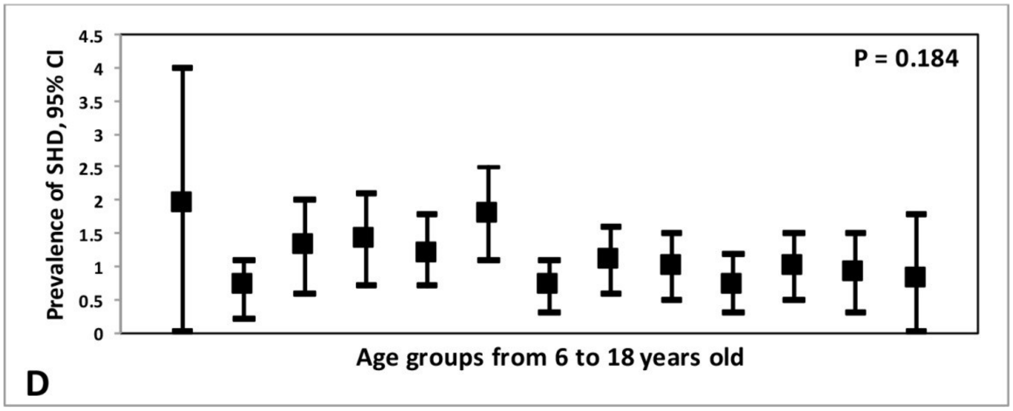

The vertical lines indicate the 95% confidence intervals for the prevalence of echocardiographic finding, and the square in the middle of the vertical line indicates the point estimate of the prevalence of echocardiographic finding in the particular age group

References

[1] Remenyi B, Wilson N, Steer A, Ferreira B, Kado J, Kumar K et al. World Heart Federation criteria for echocardiographic diagnosis of rheumatic heart disease--an evidence-based guideline. Nat Rev Cardiol 2012;9:297-309.

[2] Mirabel M, Bacquelin R, Tafflet M, Robillard C, Huon B, Corsenac P et al. Screening for rheumatic heart disease: evaluation of a focused cardiac ultrasound approach. Circ Cardiovasc Imaging 2015;8.

[3] Maron BJ, Friedman RA, Kligfield P, Levine BD, Viskin S, Chaitman BR et al. Assessment of the 12-lead electrocardiogram as a screening test for detection of cardiovascular disease in healthy general populations of young people (12-25 years of age): a scientific statement from the American Heart Association and the American College of Cardiology. J Am Coll Cardiol 2014;64:1479-514.

[4] Hancock EW, Deal BJ, Mirvis DM, Okin P, Kligfield P, Gettes LS et al. AHA/ACCF/HRS recommendations for the standardization and interpretation of the electrocardiogram: part V: electrocardiogram changes associated with cardiac chamber hypertrophy: a scientific statement from the American Heart Association Electrocardiography and Arrhythmias Committee, Council on Clinical Cardiology; the American College of Cardiology Foundation; and the Heart Rhythm Society: endorsed by the International Society for Computerized Electrocardiology. Circulation 2009;119:e251-61.

[5] Rautaharju PM, Surawicz B, Gettes LS, Bailey JJ, Childers R, Deal BJ et al. AHA/ACCF/HRS recommendations for the standardization and interpretation of the electrocardiogram: part IV: the ST segment, T and U waves, and the QT interval: a scientific statement from the American Heart Association Electrocardiography and Arrhythmias Committee, Council on Clinical Cardiology; the American College of Cardiology Foundation; and the Heart Rhythm Society: endorsed by the International Society for Computerized Electrocardiology. Circulation 2009;119:e241-50.

[6] Kligfield P, Gettes LS, Bailey JJ, Childers R, Deal BJ, Hancock EW et al. Recommendations for the standardization and interpretation of the electrocardiogram: part I: the electrocardiogram and its technology a scientific statement from the American Heart Association Electrocardiography and Arrhythmias Committee, Council on Clinical Cardiology; the American College of Cardiology Foundation; and the Heart Rhythm Society endorsed by the International Society for Computerized Electrocardiology. J Am Coll Cardiol 2007;49:1109-27.

[7] Ackerman MJ, Priori SG, Willems S, Berul C, Brugada R, Calkins H et al. HRS/EHRA expert consensus statement on the state of genetic testing for the channelopathies and cardiomyopathies this document was developed as a partnership between the Heart Rhythm Society (HRS) and the European Heart Rhythm Association (EHRA). Heart Rhythm 2011;8:1308-39.
